# Galactocele Following Aesthetic Breast Augmentation: Diagnosis, Management, and Prevention

**DOI:** 10.1093/asjof/ojaf001

**Published:** 2025-01-10

**Authors:** Hiba El Hajj, Dollen Eid, Roland Tohme, Fadi Sleilati

## Abstract

Galactorrhea and galactocele are rare complications following breast augmentation. These conditions can lead to significant patient discomfort and require careful management. This article aims to report 3 cases of galactocele that developed after aesthetic breast augmentation, proposing approaches for diagnosis, management, and prevention of galactocele. Three patients who underwent breast augmentation presented with galactocele. Clinical evaluations included aspiration of fluid collections, imaging studies, and laboratory tests to rule out infections and hormonal imbalances. Surgical interventions were tailored to each case based on patient preferences and clinical findings. In Case 1, a 40-year-old female experienced bilateral swelling 1-month postsurgery. Despite aspiration, symptoms recurred, leading to surgical revision and drainage. The implants were removed at her request. In Case 2, a 37-year-old female developed a unilateral galactocele 3 months postaugmentation after starting Norethisterone. Ultrasound-guided aspiration and surgical drainage were performed, with the implants kept in place. In Case 3, a 36-year-old female presented with wound dehiscence and lactescent discharge 7 months postaugmentation. Emergency surgery was needed for fluid evacuation, but worsening symptoms led to implant removal and drainage of bilateral galactoceles. Effective management of galactocele and galactorrhea postbreast augmentation requires a personalized approach, addressing both clinical presentations and patient-specific factors. Further awareness of these complications is essential for optimizing patient outcomes.

**Level of Evidence: 5 (Diagnostic):**

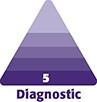

Galactorrhea and galactocele are relatively rare complications, occurring in ∼0.36% of breast augmentations following augmentation mammoplasty.^1^ Despite their rarity, these conditions can present significant challenges for affected patients. Galactorrhea, characterized by abnormal milk secretion unrelated to childbirth or lactation,^2^ and galactocele, a milk-filled cyst that can occur in the breast tissue,^3^ have been documented as postsurgical issues in a limited number of cases. These complications can arise because of hormonal imbalances and/or ductal changes induced by surgery, which may disrupt the normal physiology of the breast.^4^

This article reports 3 cases of galactocele occurring after aesthetic breast augmentation. All patients involved in this study have signed informed consent forms that explicitly grant permission to the authors to use and share their images for medical, educational, and research purposes. Because all patients underwent surgery in a private practice, approval from an institutional review board or ethics committee was not obtained. The study adhered to the principles set forth in the Declaration of Helsinki.

## CASE 1

A 40-year-old female presented with recurrent bilateral breast swelling following aesthetic breast augmentation. She has a history of 5 pregnancies and 4 deliveries, including twins, 2 boys, and a girl. Her last delivery was 1 year and 8 months ago. She breastfed for 6 months and received cabergoline for 2 days to suppress lactation. From time to time, she noted occasional milk discharge from her nipples on breast squeezing.

One year after she stopped breastfeeding, the patient underwent bilateral aesthetic breast augmentation through a periareolar incision with an implant placed in a dual plane Type 1. One month later, she began experiencing moderate swelling in her breasts. The patient has no significant medical history and is not on any medications. She has no history of similar episodes, polycystic ovary syndrome (PCOS), hepatic insufficiency, thyroid dysfunction, or adrenal insufficiency. There was no known use of contraceptives, antidepressants, or neuroleptics before this event.

She was thought to have a seroma. An ultrasound revealed bilateral fluid collections around her breast implants. During an ultrasound-guided aspiration, we noticed that the fluid was lactescent and proceeded to evacuate a total of 350 mL of it while retaining the implants. The culture of the fluid did not identify any organisms, histopathology ruled out the presence of anaplastic large-cell lymphoma (ALCL), and SUDAN IV staining was positive, which is consistent with the presence of milk.

Two weeks later, the patient developed similar swelling in both breasts and came to our office for further management. On physical examination, she presented with bilateral breast swelling and mild tenderness on palpation but no fever, erythema, or signs of infection.

A second ultrasound-guided aspiration also revealed a white fluid, which was sent for a second culture and histopathological examination. The results were again negative for infection, ALCL, and SUDAN IV positive. Complete blood count (CBC), prolactin (PRL) level, C-reactive protein, thyroid-stimulating hormone (TSH), and beta-hCG were all negative.

We decided to proceed with a new operation through her existing periareolar incision scar in the operating room. Upon opening the implant capsule, a significant amount of lactescent fluid was encountered, with nearly 700 mL drained from each breast. The pocket was irrigated with Adams’ solution: 50,000 U bacitracin, 1 g cefazolin, and 80 mg gentamicin.^5^

As the patient did not wish to risk any further complications, the implants were removed. A capsulotomy was performed to facilitate cavity obliteration. Drains were placed in the pockets, and the patient was discharged the same day.

The final cultures were negative, as was the histopathological examination. The drains were removed 2 days postoperatively when the discharge amount was <30 mL/day for 2 consecutive days.

## CASE 2

A 37-year-old monoparous female presented with a unilateral galactocele that appeared ∼3 months following breast augmentation surgery with an inferior inframammary fold incision, dual plane Type 3, using a 295 cc breast implant bilaterally. To achieve symmetry in size and reduce the projection of the left breast, a retroareolar glandular disk—a section of glandular breast tissue located directly behind the areola—was removed. The patient had been taking Norethisterone for spotting for 10 days, starting 2 weeks before the onset of the galactocele. She had a C-section 5 years ago when she breastfed for 3 to 4 months. She has no history of similar episodes, PCOS, hepatic insufficiency, or adrenal insufficiency. Additionally, there is no known use of contraceptives, antidepressants, or neuroleptics before this event.

**Figure 1.**
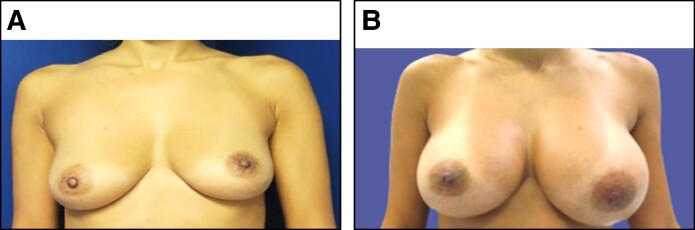
(A) Preoperative image of the 37-year-old female from Case 2. (B) Three-month postoperative image showing galactocele formation in the same patient. This patient had completed a 10-day course of Norethisterone 2 weeks before the galactocele developed.

On physical examination, the galactocele was unilateral (Figure 1), and the patient reported no signs of infection such as pain or erythema. Paraclinical examinations revealed normal results for CBC, C-reactive protein, beta-hCG, and TSH. PRL levels were 22.36 ng/mL, which is within the normal range of 5 to 25 ng/mL. Additionally, beta-hCG, culture, and histopathology results were negative except for SUDAN IV dye.

On ultrasound, there was a significant left periprosthetic effusion with a heterogeneous echostructure containing nonspecific mobile hyperechoic structures.

**Figure 2.**
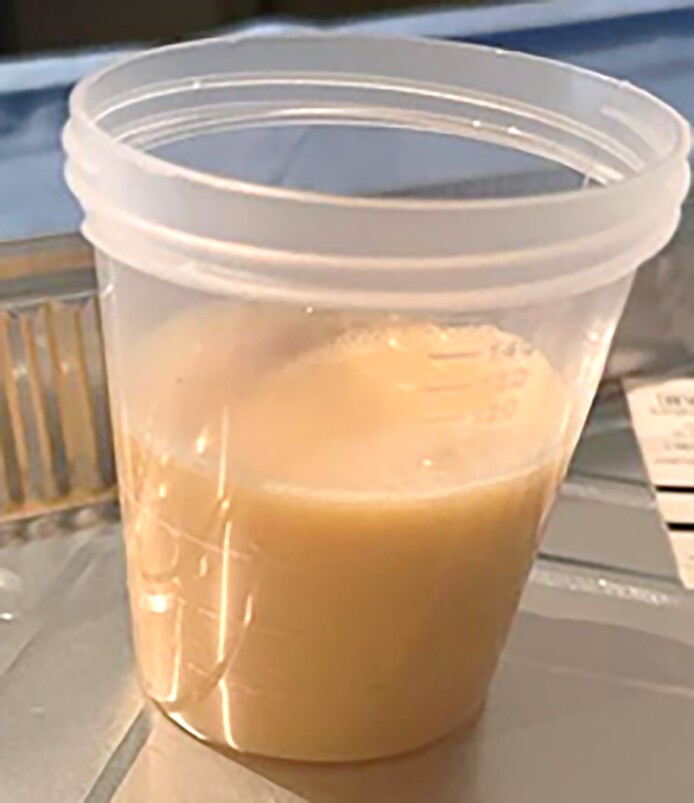
Lactescent fluid aspirated from the left breast of the 37-year-old female patient in Case 2, performed under ultrasound guidance.

**Figure 3.**
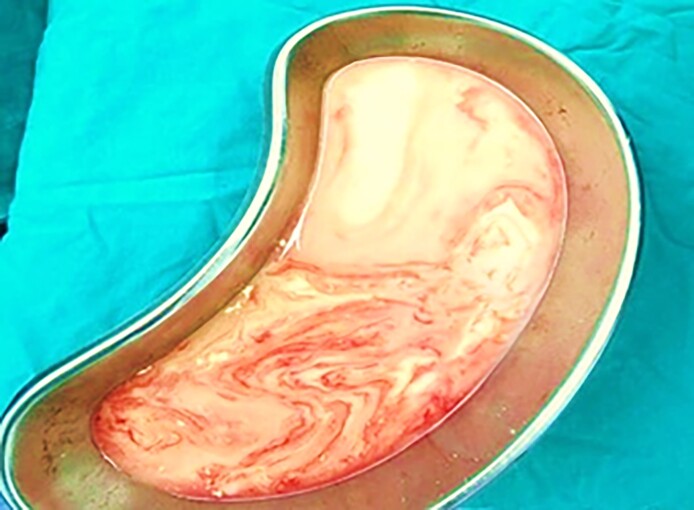
Container holding the fluid drained from the left breast of the 37-year-old female in Case 2.

An ultrasound-guided aspiration was performed using an 18 G needle, retrieving 40 mL of lactescent fluid (Figure 2). The pathology analysis revealed predominantly macrophagic inflammatory material with no suspicious malignant cells detected. Management involved draining 300 mL of fluid (Figure 3). The pocket was irrigated with 1 L of saline and Adams’ solution, and the prosthesis was retained. No additional complications followed.

## CASE 3

**Figure 4.**
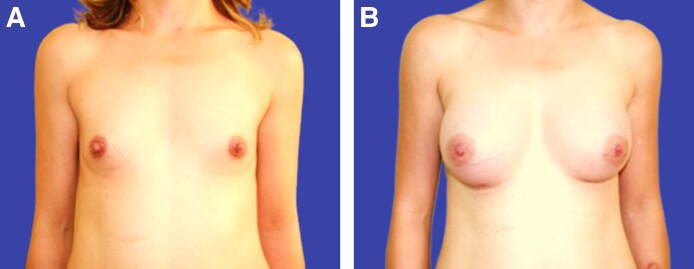
(A) Preoperative image of the breast from the 36-year-old female in Case 3. (B) The same breast 6 weeks postoperatively from the same patient.

A 36-year-old female presented ∼7 months after undergoing bilateral breast augmentation with breast pain and galactorrhea. The implants, 260 cc textured prostheses, were placed in a dual plane pocket using inframammary fold incisions (Figure 4). Her medical history includes 4 pregnancies, all delivered through cesarean section, with the last one occurring 3 years ago when she breastfed her third child for 6 months. She has no history of similar episodes, PCOS, hepatic insufficiency, thyroid dysfunction, or adrenal insufficiency. Additionally, there is no known use of contraceptives, antidepressants, or neuroleptics before this event.

On physical examination, we observed a dehiscence of the left breast wound with exposure of the prosthesis and a lactescent discharge. The right breast was tense and tender to palpation but showed no signs of inflammation. Laboratory tests, including CBC, beta-hCG, PRL, C-reactive protein, TSH, T3, and T4, were all within normal limits.

The patient was operated on urgently. A bacteriological sample was taken from the fluid. The pocket was irrigated thoroughly, and the implants were kept in place. The patient was started on antibiotics. Drains were placed; each produced 300 mL. A bacteriological sample was taken, and the patient was started on antibiotics.

**Figure 5.**
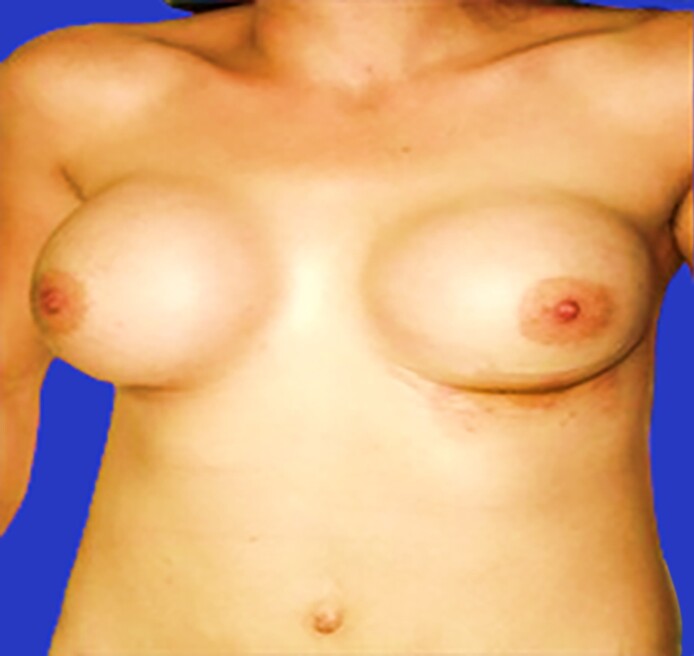
Bilateral galactocele observed 7 months postoperatively from the 36-year-old female in Case 3 following postoperative hormonal injections.

**Figure 6.**
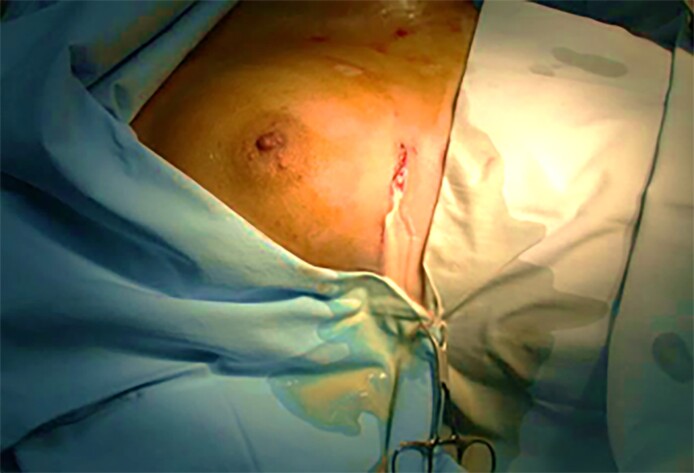
White discharge drained from the 36-year-old female's right breast through a new inframammary incision, created along the site of the previous inframammary incision from Case 3.

Five days later, the patient returned with worsening symptoms. Given the presence of significant bilateral lactescent discharge and breast swelling (Figure 5), the patient was operated on for removal of the breast implants, overall evacuation of ∼500 mL of galactoceles during the final procedure, placement of drains, and administration of cabergoline (Figure 6). Upon further questioning, the patient disclosed that she had received postoperative hormonal injections, reportedly intended to enhance breast sensitivity and libido.

## DISCUSSION

This case series highlights 3 instances of galactocele following aesthetic breast augmentation, a rare but significant complication. Because an elective procedure aimed at enhancing appearance and boosting self-esteem, breast augmentation comes with high patient expectations and minimal tolerance for complications. Thus, managing postoperative issues effectively, namely galactocele, is crucial for maintaining patient satisfaction. Galactocele, though uncommon, can compromise the aesthetic result and introduce risks such as implant loss and the need for additional surgeries, leading to emotional distress and dissatisfaction. Effective prevention and management are essential for the physical and psychological well-being of patients.

### Case Analysis

In the first case, several risk factors contributed to the development of a galactocele. A notable factor is the patient's obstetric history. A higher number of pregnancies is associated with an increased risk of these complications.^6^ Additionally, the patient underwent surgery 1 year after stopping breastfeeding, a period that may still pose a risk for galactocele development.^7^ The periareolar incision used in the surgery is specifically associated with a higher risk of ductal obstruction and galactocele formation. This increased risk may potentially lead to complications, such as implant contamination and infection.^8^ A review by Sharma and Basu found that 50% of patients with galactocele complications had undergone periareolar incisions.^7^

The second case involved the use of Norethisterone, a progestin medication widely used for contraception, postmenopausal hormone therapy, and the treatment of abnormal uterine bleeding and endometriosis.^9^ Norethisterone is known to increase galactocele risk.^10^ A systematic review indicates that oral contraceptives can also be a contributing factor to galactorrhea and galactocele development.^7^ Additionally, the removal of a retroareolar glandular disk from the left breast increased the risk of this complication, alongside other contributing factors.

The third case highlights the combination of multiple risk factors, including a high number of pregnancies and postoperative hormonal injections. The case also involved an inframammary fold incision, which was reported to be a factor in 12% of similar cases.^7^ These factors contributed to a complex postoperative course and galactocele formation.

### Management Strategy

A proposed treatment algorithm categorizes patients into high-, moderate-, or low-risk groups based on incision site and implant insertion plane.

For periareolar or subglandular implants: For patients with postoperative galactorrhea, breast ultrasonography is recommended. If fluid collection is detected, the patient is categorized as high risk, requiring implant removal and bromocriptine therapy. If no fluid is observed, further management depends on PRL levels.^11^

For inframammary or transaxillary implants, PRL levels should be checked. Elevated levels categorize the patient as intermediate risk, necessitating bromocriptine treatment and a brain MRI. A brain MRI is performed to rule out a PRL-secreting tumor, which may be causing central hyperprolactinemia. This imaging study helps identify any abnormalities in the pituitary gland or surrounding structures that could be contributing to elevated PRL levels. Normal PRL levels suggest low risk and are treated with reassurance and observation, including repeat measurements of serum PRL levels every 3 to 6 months.^11^

For postoperative milk discharge, the SUDAN IV test can help confirm the presence of milk. SUDAN IV is a lipid-soluble dye that binds specifically to lipids, allowing for their visualization under a microscope. By using SUDAN IV, we were able to assess the lipid profile of the discharge in our 3 cases, confirming that its composition was consistent with milk.

If galactocele is suspected, breast ultrasound and drainage are indicated, followed by serum PRL measurement and appropriate treatment. Discontinuing oral contraceptives and tapering off medications when resolved are also recommended.

Another treatment algorithm recommends assessment of serum PRL levels. If PRL is below 100 ng/mL, start bromocriptine at 5 mg/day for 7 days, then re-evaluate. Discontinue oral contraceptives if applicable and taper off the drug when resolved. If PRL is above 100 ng/mL, perform an MRI, check TSH levels, and refer to an endocrinologist for further evaluation.^12^

### Prevention and Monitoring

Primary prevention of galactocele requires a thorough preoperative evaluation of risk factors, including a patient's medical history, hormonal status, incision site, and implant placement. A carefully taken medical history is very important in preoperative preparation and patient selection. There are no guidelines on how long females should wait after pregnancy or breastfeeding before undergoing breast augmentation. However, expert consensus typically suggests waiting 3 to 6 months to allow milk production to cease and for the breasts to return to their “new normal” size. Given that a recent history of breastfeeding (within the past year) may increase the risk of galactorrhea or galactocele,^7^ waiting up to 1 year after delivery and the cessation of breastfeeding is considered a reasonable approach.^13^

Several pharmacological agents have been associated with an increased risk of galactorrhea, and their management in the perioperative period is crucial. As seen in previously reported cases,^10,11,14^ galactorrhea may also be promoted by stopping oral contraceptive pills a few days before the procedure; the estrogen hormone concentration is no longer sufficient to block the action of PRL. At the same time, a preoperative history of oral contraceptive use may increase the risk of galactorrhea after breast surgery.^10^ Unfortunately, there is currently insufficient evidence to determine the optimal time to discontinue oral contraceptives before surgery to minimize the risk of galactocele. Timing may also depend on individual risk factors, such as hormonal sensitivity, surgical complexity, and previous breastfeeding history.

Other medications known to promote galactorrhea are antiemetics like metoclopramide^15^ and some neuroleptics and antidepressants^13,16^ and antihypertensive drugs (verapamil, methyldopa, and reserpine).^15^ Whenever possible, all inducing medications should be discontinued before the operation. The recommended period to stop these medications is equivalent to 6 half-lives of the drug’s active substance.^13^ Postoperatively, close monitoring for signs of galactocele, such as unilateral breast swelling, pain, or discharge is essential. Early use of bromocriptine in patients with elevated PRL levels, and patient education on signs and symptoms are crucial steps.^17^ To reduce the risk of developing a galactocele, it is important to advise patients to avoid nipple stimulation during the postoperative period, educate them about medications that can increase their risk—such as oral contraceptives, hormone therapy, and antipsychotics—and encourage them to report any upcoming symptoms early.

By implementing these strategies, surgeons can significantly reduce the risk of galactocele and improve overall outcomes and patient satisfaction following breast augmentation.

**Figure 7.**
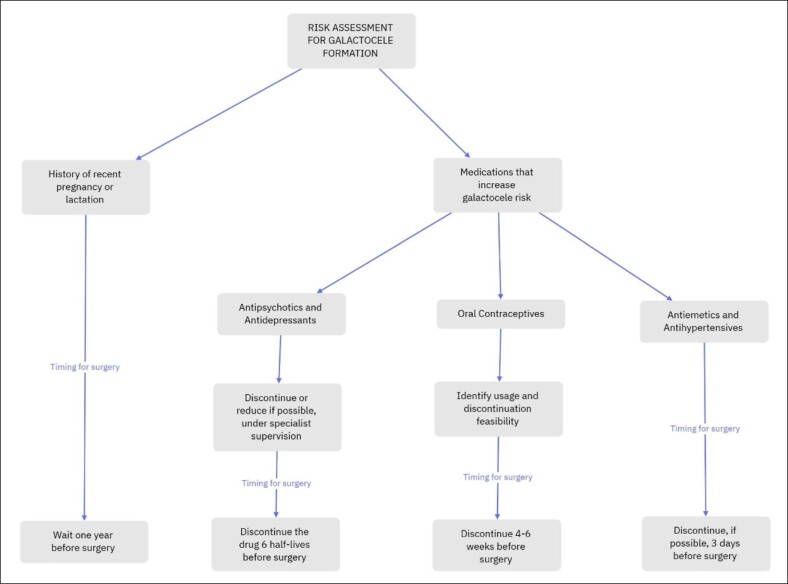
Risk Assessment for Galactocele Formation: this flowchart provides a structured approach for assessing patients’ risk of galactocele formation before surgery. Patients with a history of recent pregnancy or lactation, or those on medications that increase galactocele risk, are advised to consider specific timing for surgery and potential medication adjustments. Recommendations include waiting 1 year postlactation, discontinuing antipsychotics, and antidepressants with specialist supervision (6 half-lives before surgery), stopping oral contraceptives 4 to 6 weeks before, and discontinuing antiemetics and antihypertensives 3 days before surgery if possible.

**Figure 8.**
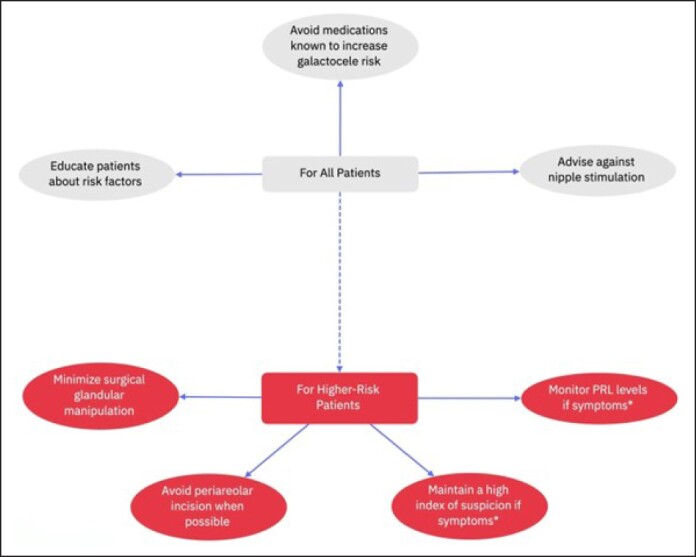
Perioperative and postoperative prevention strategies for galactocele formation: this flowchart outlines preventive measures for all patients undergoing breast augmentation, as well as additional precautions for those at higher risk of galactocele formation. Patients are considered at higher risk of galactocele if they are on any medication known to increase galactocele risk and cannot safely discontinue it, or if they have a recent history of pregnancy or lactation. For all patients: educate patients on galactocele risk factors, avoid initiating medications known to increase the risk, and advise against nipple stimulation postoperatively. For higher risk patients: Consider avoiding periareolar incisions and minimizing glandular manipulation during surgery. In the postoperative period, monitor PRL levels and maintain a high index of suspicion for galactocele if symptoms, such as nipple discharge or breast swelling, are reported. *Symptoms: Nipple discharge, breast swelling, or tenderness that may indicate early signs of galactocele formation.

Patients at high risk of developing galactoceles, such as those with risk factors that cannot be modified—like a high number of pregnancies or the use of antipsychotics that cannot be discontinued—might benefit from more frequent monitoring through PRL level assessments and increased awareness of their condition. More research is needed to determine whether these patients should receive dopamine agonists as a primary preventive measure. We developed a flowchart for risk assessment and patient education to guide preventive measures in the postoperative period (Figure 7). This flowchart is intended to help identify at-risk patients and provide tailored educational strategies. Additionally, a second flowchart outlines a stepwise management approach during surgery and postoperatively based on individual patient risk levels (Figure 8), ensuring appropriate interventions are taken depending on the assessed risk. However, further studies are required to assess whether to dose the PRL in patients under specific medications, especially that in 38%, galactocele can happen with normal PRL dosage.^7^

## CONCLUSIONS

These cases underscore the various presentations and management challenges associated with galactocele and galactorrhea after aesthetic breast augmentation. Although rare, the occurrence of galactocele can significantly impact patient satisfaction and the overall success of the procedure. Through comprehensive risk factor assessment and a tailored treatment approach, it is possible to minimize the incidence of this complication and ensure timely and effective management when it arises. Prevention, early identification, and targeted intervention are keys to achieving optimal outcomes for patients undergoing breast augmentation.
